# NFBD1/MDC1 Is Phosphorylated by PLK1 and Controls G2/M Transition through the Regulation of a TOPOIIα-Mediated Decatenation Checkpoint

**DOI:** 10.1371/journal.pone.0082744

**Published:** 2013-12-11

**Authors:** Kiyohiro Ando, Toshinori Ozaki, Toru Hirota, Akira Nakagawara

**Affiliations:** 1 Division of Biochemistry and Innovative Cancer Therapeutics, Chiba Cancer Center Research Institute, Chiba, Japan; 2 Division of Clinical Oncology Research, Shonan Kamakura General Hospital, Kanagawa, Japan; 3 Laboratory of Anti-tumor Research, Chiba Cancer Center Research Institute, Chiba, Japan; 4 Department of Experimental Pathology, Cancer Institute of the Japanese Foundation for Cancer Research (JFCR), Tokyo, Japan; German Cancer Research Center, Germany

## Abstract

Although it has been established that nuclear factor with BRCT domain 1/ mediator of the DNA damage checkpoint protein 1 (NFBD1/MDC1) is closely involved in DNA damage response, its possible contribution to the regulation of cell- cycle progression is unclear. In the present study, we have found for the first time that NFBD1 is phosphorylated by polo-like kinase 1 (PLK1) and has an important role in G2/M transition. Both NFBD1 and PLK1 are co-expressed in cellular nuclei throughout G2/M transition, and binding assays demonstrated direct interaction between NFBD1 and PLK1. Indeed, *in vitro* kinase reactions revealed that the PST domain of NFBD1 contains a potential amino acid sequence (845-DVTGEE-850) targeted by PLK1. Furthermore, enforced expression of GFP-PST but not GFP-PST(T847A) where threonine at 847 was substituted by alanine inhibited the phosphorylation levels of histone H3, suggesting a defect of M phase entry. Because PLK1 has been implicated in promoting the G2/M transition, we reasoned that overexpressed PST might serve as a pseudosubstrate for PLK1 and thus interfere with phosphorylation of endogenous PLK1 substrates. Interestingly, siRNA-mediated knockdown of NFBD1 resulted in early M phase entry and accelerated M phase progression, raising the possibility that NFBD1 is a PLK1 substrate for regulating the G2/M transition. Moreover, the constitutive active form of PLK1(T210D) overcame the ICRF-193-induced decatenation checkpoint and inhibited the interaction between NFBD1 and topoisomerase IIα, but kinase-deficient PLK1 did not. Based on these observations, we propose that PLK1-mediated phosphorylation of NFBD1 is involved in the regulation of G2/M transition by recovering a decatenation checkpoint.

## Introduction

 Upon DNA damage, ataxia-telangiectasia mutated (ATM) protein kinase is activated through its phosphorylation, and then histone variant H2AX is phosphorylated (γ-H2AX) by the activated form of ATM to form nuclear foci at DNA double-strand break sites. This ATM-regulated nuclear event is followed by recruitment of the multifunctional MRE11-RAD50-NBS1 complex onto sites of DNA damage to facilitate DNA repair, which is mediated by the checkpoint mediator NFBD1/MDC1 (henceforth NFBD1) [1-3]. 

 NFBD1 is a large nuclear phospho-protein containing NH_2_-terminal forkhead-associated (FHA), central proline/serine/threonine-rich (PST), and COOH-terminal tandem repeats of BRCA1 carboxyl terminus (BRCT) domains. Among them, the BRCT domain contributes to the interaction with phosphopeptides. Several lines of evidence suggest that the BRCT domain of NFBD1 acts as a phosphoserine-binding pocket and is involved in the interaction with γ-H2AX [4,5]. Additionally, NFBD1 is one of the substrates of ATM [1,2]. Indeed, *NFBD1*-deficient mice display chromosome instability, DNA repair defects, and radiation sensitivity [6]. Therefore, NFBD1 has been implicated in the recognition and repair of DNA double-strand breaks through its rapid recruitment of γ-H2AX in response to DNA damage [3]. Accumulating evidence has revealed that NFBD1 is a critical upstream mediator in the cellular response to double-strand breaks and is associated with cell-cycle arrest induced by the activation of intra-S and G2/M transition checkpoints [1,3]. Therefore, these studies indicate that NFBD1 might contribute to cell-cycle regulation. Alternatively, Luo et al. reported that the interaction between NFBD1 and TOPOIIα is required for activation of the decatenation checkpoint, which is induced by ICRF-193, a catalytic TOPOIIα inhibitor, and causes cell-cycle arrest at the G2 phase in mammalian cells [7]. However, the functional relationship between NFBD1 and the essential cell-cycle regulators of G2/M transition, such as mitotic kinases, are still unknown.

 Polo-like kinase (PLK) was initially identified in *Drosophila*, and mammalian cells express at least four distinct PLKs including PLK1–4 [8]. Among them, PLK1 is the most extensively studied and plays a critical role in the regulation of entry into mitosis as well as exit from mitosis, including mitotic promoting factor activation [9,10], centrosome maturation, microtubule nucleation, facilitating kinetochore assembly, regulating spindle assembly checkpoint, and completion of cytokinesis. Among the multiple roles of PLK1 in cell division, kinase activity has emerged as an attractive target in anti-proliferative cancer therapy [11,12]. Consequently, anti-sense oligonucleotides, small interfering RNA (siRNA) or several potent and specific small molecule inhibitors against PLK1 have been developed over the past 2 years, and some have been tested in clinical trials [13,14]. Paradoxically, because kinase activity of PLK1 must be tightly regulated during cell division, it might be hypothesized that either up-regulated or down-regulated PLK1 could induce mitotic defects that result in aneuploidy and tumorigenesis. In accordance with this idea, mice heterozygous for plk1 developed tumors at threefold greater frequency than their wild-type counterparts, suggesting that plk1 functions as a haploinsufficient tumor suppressor. Therefore, using PLK1 as a therapeutic target may not be as straightforward as previously thought [15]. PLK1 inhibitors may be dependent on the genetic status of each tumor type, for example, containing a mutation of tumor suppressor p53, and may require validation for each tumor type [16].

Regarding G2/M transition, plk1 contributes to the activation of cdk1 through phosphorylation of cdk1-activating phosphatase cdc25 and thereby forms a positive feedback loop to accelerate M phase entry in *Xenopus laevis* [17-19]. In contrast, van Vugt et al. demonstrated that PLK1 is dispensable for the G2/M transition in human cells [20]. In support of this hypothesis, silencing of PLK1 or expression of a dominant-negative PLK1 mutant resulted in mitotic arrest [21-23]. However, recent work in mammalian cells has revealed that phosphorylation of PLK1 in the activation loop (T210) by aurora A (AURKA) leads to a burst of PLK1 activity at the G2/M transition and efficient entry into mitosis [24,25]. Therefore, the essential role of PLK1 in G2/M transition has been controversial. 

In the present study, we have found for the first time that PLK1-mediated phosphorylation of NFBD1 plays a pivotal role in the regulation of G2/M transition in mammalian cells, and hyper-phosphorylation by PLK1 might contribute to genomic instability and tumorigenesis. 

## Results

### NFBD1 and PLK1 proteins are coexistent in G2/M phase of cell cycle

**Figure 1:**
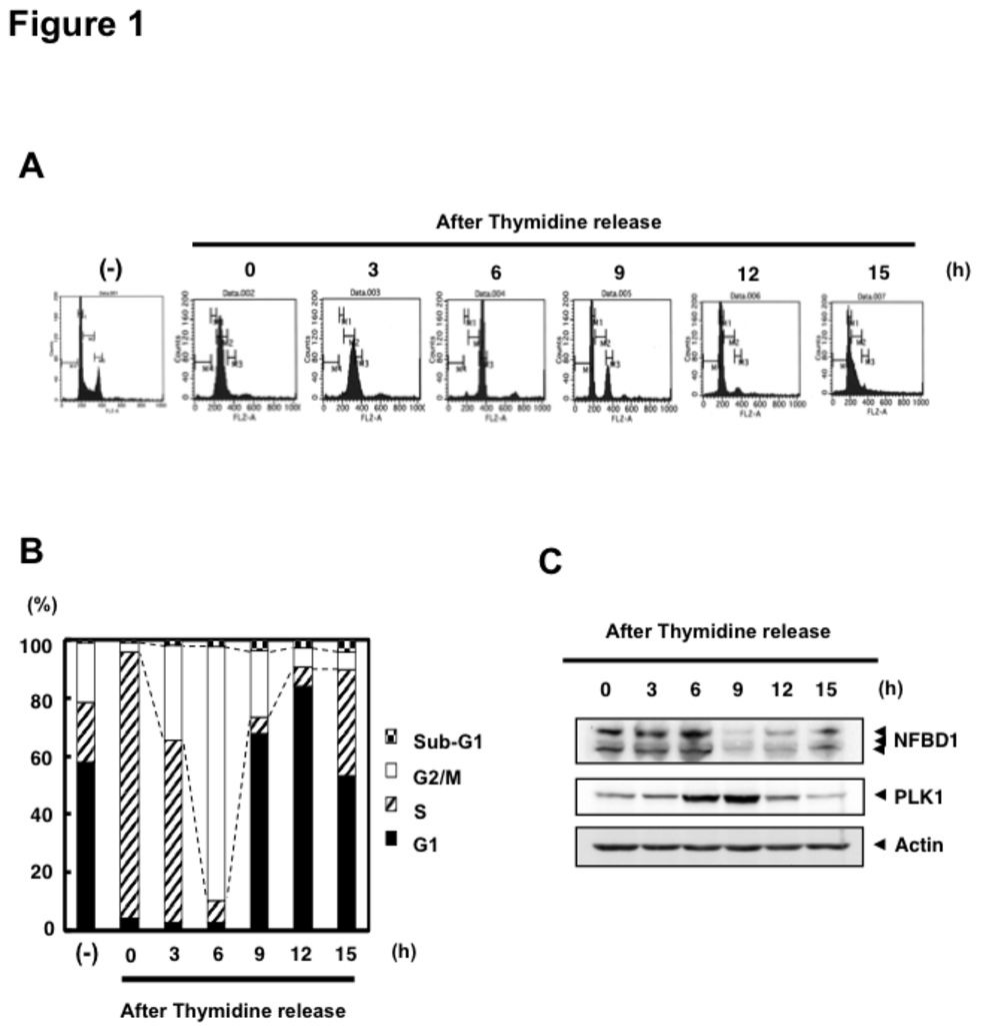
*NFBD1* and PLK1 are both expressed in the G2/M phase and form a protein complex. (A) HeLa cells were double-thymidine blocked at the late G1 phase and then released into fresh medium. At the indicated times after release, cells were stained with propidium iodide (PI) and analyzed by FACS. (B) Schematic representation of cell-cycle distributions under the abovementioned experimental conditions. (C) Western blot analysis. At the indicated time after release, whole cell lysates were prepared and immunoblotted against anti-NFBD1 (first panel), anti-PLK1 (second panel), or anti-actin (third panel) antibody. Immunoblotting for actin is shown as a control for protein loading.

 Xu et al. have shown that NFBD1 protein levels were low in S phase and higher in cell populations enriched for G2/M and G1 in human cervical carcinoma HeLa S3 cells [26]. To access the protein levels of NFBD1 and PLK1 during cell-cycle progression, HeLa cells were double-thymidine blocked and then released into fresh medium to allow their progression through the cell cycle. At the indicated times after release from the double-thymidine block, floating and attached cells were harvested and stained with propidium iodide; their cell-cycle distributions were examined by FACS. As shown in Figure 1A and 1B, cells were synchronized in the late G1 phase at 0 h after the second release and began to enter into the G2 phase through the S phase at 3 h after the release. As judged from the clear accumulation of cells with 4N DNA content at 6 h after the release, the majority of cells entered into G2 or M phases. Nine hours after the release, over 60% of the cells passed through the M phase. Under these experimental conditions, whole cell lysates were prepared at the indicated times after the release and analyzed by immunoblotting for the protein levels of PLK1 and NFBD1. As shown in Figure 1C, the protein levels of PLK1 were dramatically increased at 6 h and peaked at 9 h after the release. On the other hand, the protein levels of NFBD1 were high until 6 h after the release. These results indicated that PLK1 and NFBD1 are coexistent in cells during the G2/M phase of the cell cycle. However, in contrast to the previous report by Xu et al., we have observed that NFBD1 protein levels were down-regulated and/or degraded in G1 phase in our experimental condition. These conflicting results might be due to the differences of epitopes recognized by these antibodies. David F Stern’s group and our group raised antibodies against N-terminus regions of NFBD1, amino acid residues 142 to 568 and 1 to 150, respectively. Since the FHA domain has been reported to receive its posttranslational modifications such as phosphorylation [27], we speculate that these modifications could affect the protein detection.

### Interaction between NFBD1 and PLK1 in cells

**Figure 2:**
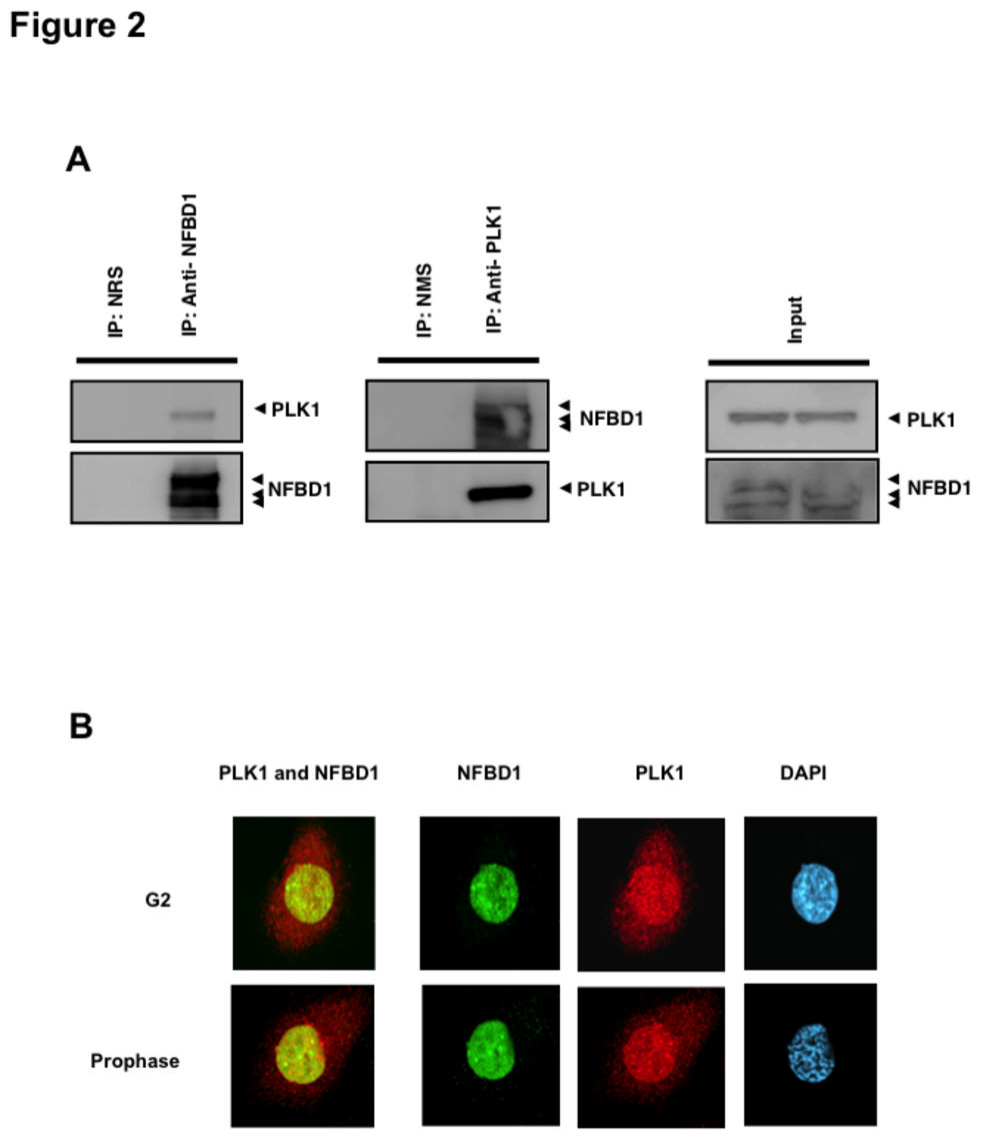
Co-immunoprecipitation and co-localization between NFBD1 and PLK1 in the G2/M phase. (A) Immunoprecipitation analysis. HeLa cells were synchronized by the double-thymidine block regimen. Six hours after the second release, whole cell lysates prepared from HeLa cells were immunoprecipitated with normal rabbit serum or with a polyclonal anti-NFBD1 antibody followed by immunoblotting with a monoclonal anti-PLK1 antibody (left panel). Reciprocal experiments using normal mouse serum and a monoclonal anti-PLK1 antibody are shown in the middle panel. (B) Indirect immunofluorescence staining. HeLa cells were synchronized by the double-thymidine block regimen. Six hours after the second release, mitotic cells were simultaneously stained with polyclonal anti-NFBD1 (green) and monoclonal anti-PLK1 antibodies (red). Merged images (yellow) indicate the co-localization of NFBD1 with PLK1. Nuclear DNA was stained with DAPI (blue).

 To investigate a possible interaction between NFBD1 and PLK1, we employed immunoprecipitation experiments. Whole cell lysates prepared from HeLa cells were immunoprecipitated with normal rabbit serum (NRS) or with polyclonal anti-NFBD1 antibody, and the anti-NFBD1 immunoprecipitates were analyzed by immunoblotting with monoclonal anti-PLK1 antibodies. As demonstrated in Figure 2A, anti-NFBD1 immunoprecipitates contained endogenous PLK1. Similarly, the reciprocal experiments demonstrated that NFBD1 is co-immunoprecipitated with PLK1. These results suggest that NFBD1 interacts with PLK1 in cultured mammalian cells. To evaluate the subcellular localization of NFBD1 and PLK1, we performed indirect immunofluorescence staining during G2 and M phase in HeLa cells. As shown in Figure 2B, we observed that in the G2 phase and the prophase before nuclear-envelope breakdown (NEBD), NFBD1 is expressed exclusively in the cell nucleus, and PLK1 is detectable in both the cell nucleus and cytoplasm. Therefore, these observations strongly suggested that NFBD1 co-localized with PLK1 in the cell nucleus throughout the G2 phase and the prophase. 

**Figure 3:**
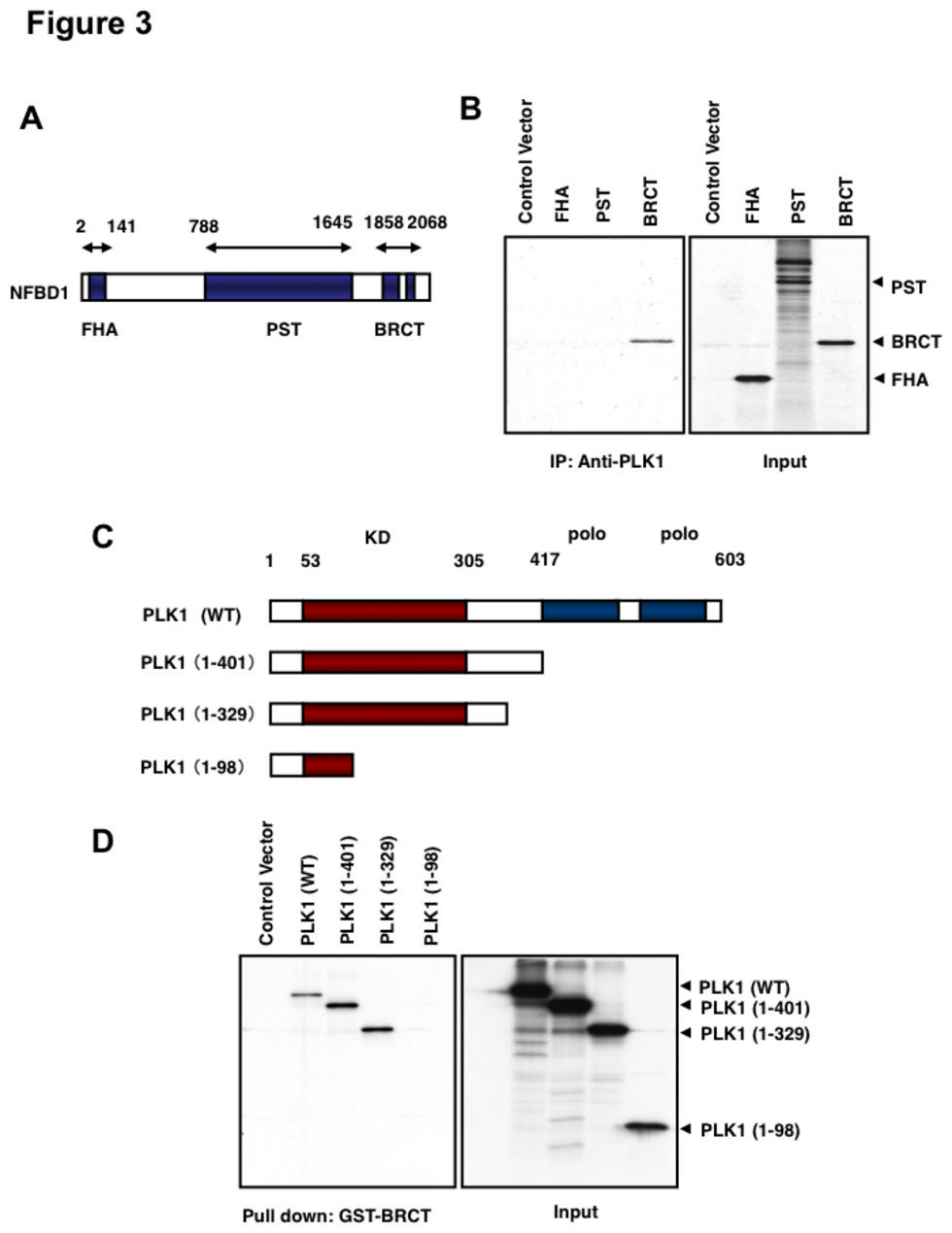
Mapping of the binding regions between NFBD1 and PLK1. (A) A schematic drawing of the structure of NFBD1 is shown. Numbers indicate the amino acid positions relative to the first Met (+1). FHA, forkhead-associated; PST, proline/serine/threonine-rich; BRCT, BRCA1 carboxyl terminus. (B) *In* *vitro* binding assay. Whole cell lysates prepared from COS7 cells transfected with FLAG-Plk1 expression plasmid were incubated with ^35^S-labeled FHA, PST, or the BRCT domain of NFBD1. Bound materials were recovered by immunoprecipitation with an anti-PLK1 antibody and analyzed by SDS-PAGE followed by autoradiography (left panel). The right panel shows 1/10 loading. (C) The structure of wild-type Plk1 and various COOH-terminal deletion mutants of PLK1. KD, kinase domain; polo, polo box domain. (D) GST pull-down assay. ^35^S-labeled wild-type PLK1 or the indicated deletion mutants of PLK1 were incubated with GST-BRCT fusion protein. After the incubation, bound proteins were recovered on glutathione Sepharose beads and separated by SDS-PAGE followed by autoradiography (left panel). The right panel shows 1/10 loading.

 To identify the region(s) of NFBD1 required for interaction with PLK1, we performed an *in vitro* pull-down assay. Whole cell lysates prepared from COS7 cells transfected with the FLAG-PLK1 expression plasmid were incubated with the indicated radiolabeled FHA, PST, or with the BRCT domain of NFBD1 and immunoprecipitated with an anti-PLK1 antibody. As clearly shown in Figure 3A and B, anti-PLK1 immunoprecipitates contained the radiolabeled BRCT domain, indicating that the extreme COOH-terminal BRCT domain is responsible for the complex formation with PLK1. Next, to map the BRCT domain-interacting regions on PLK1, we purified GST-BRCT and performed an *in vitro* pull-down assay to investigate the interaction with various PLK1 deletion mutants. As shown in Figure 3C and 3D, the amino acid sequence comprising residues 99–329 on PLK1, which included the kinase domain, appeared to be the BRCT-binding domain.

### PLK1 phosphorylates NFBD1 *in vitro*

**Figure 4:**
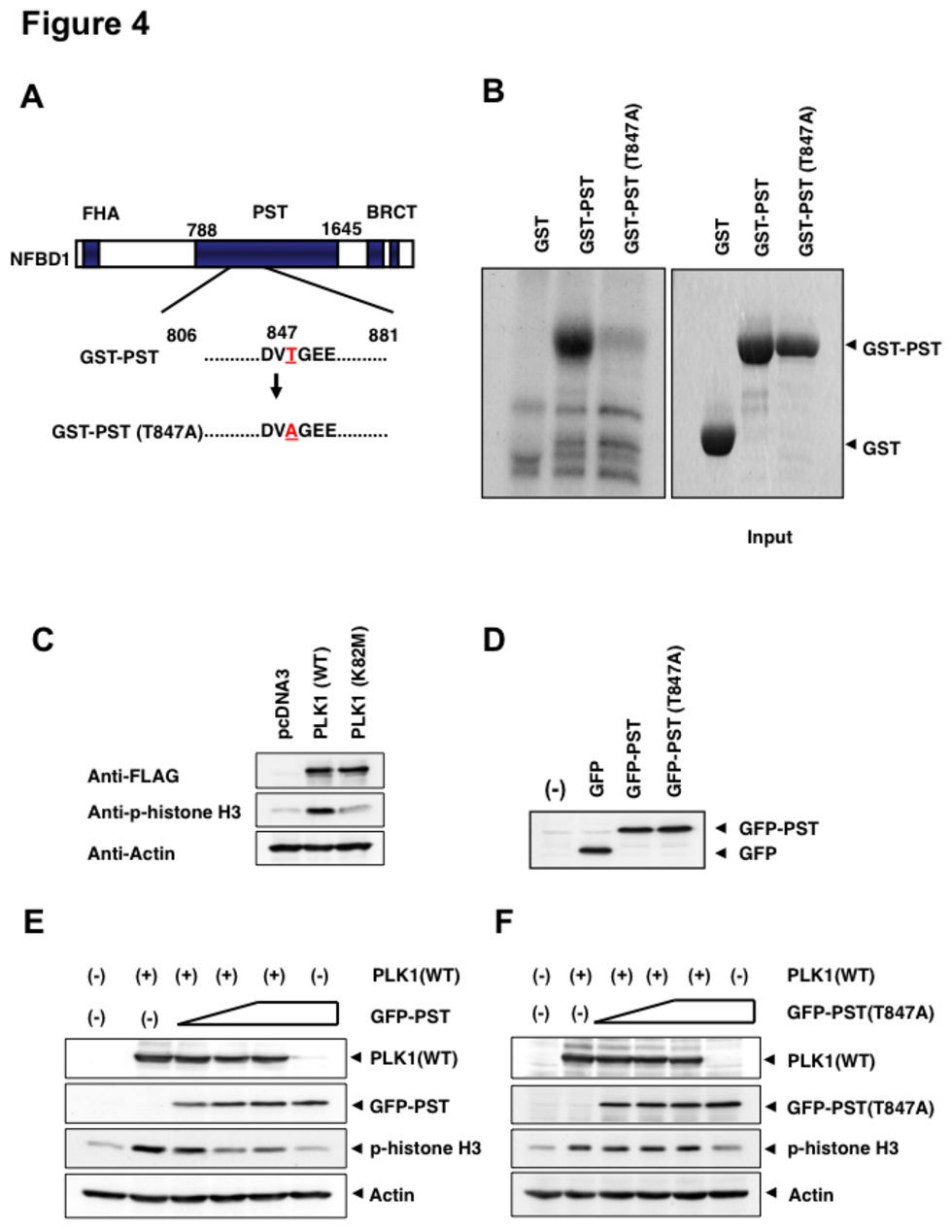
PLK1-mediated phosphorylation of NFBD1 is essential for the M phase entry. (A) Schematic representation of PST domains of wild-type NFBD1 and mutant form of NFBD1 termed GST-PST(T847A). (B) *In* *vitro* kinase reaction. GST, GST-PST, or GST-PST(T847A) were purified using glutathione Sepharose beads (right panel) and incubated with purified PLK1 in the presence of [γ-^32^P]ATP. The reaction mixtures were analyzed by SDS-PAGE followed by autoradiography (left panel). (C) Enforced expression of PLK1 but not the kinase-deficient mutant form of PLK1 [PLK1(K28M)] induces phospho-histone H3. HeLa cells were transiently transfected with the indicated expression plasmids. Forty-eight hours after transfection, whole cell lysates were prepared and immunoblotted with the indicated antibodies. (D) Expression of GFP-PST and GFP-PST(T847A). HeLa cells were transiently transfected with the expression plasmids. Forty-eight hours after transfection, whole cell lysates were prepared and immunoblotted with an anti-GFP antibody. (E and F) GFP-PST but not GFP-PST(T847A) inhibits the phosphorylation of histone H3. HeLa cells were transiently transfected with the expression plasmid for FLAG-PLK1 alone or FLAG-PLK1 and increasing amounts of GFP-PST (E) or GFP-PST(T847A) (F). Forty-eight hours after transfection, whole cell lysates were prepared and analyzed by immunoblotting with the indicated antibodies.

 Because the kinase domain of Pkl1 interacts with the BRCT domain of NFBD1, we then asked whether Pkl1 could phosphorylate NFBD1. For this purpose, we searched for the consensus amino acid sequence(s) of NFBD1 targeted by Plk1 [28,29] and found a putative amino acid sequence (845-DVTGEE-850) within the PST domain of NFBD1 possibly targeted by PLK1 (Figure 4A). We then generated GST fusion constructs encoding the wild-type PST domain termed GST-PST and the mutant form of the PST domain where Thr was substituted by Ala (845-DVAGEE-850) termed GST-PST(T847A). GST and these GST fusion proteins were purified by glutathione Sepharose beads (right panel of Figure 4B) and subjected to the *in vitro* kinase reaction using purified PLK1. As clearly shown in the left panel of Figure 4B, GST-PST but not GST-PST (T847A) was phosphorylated by PLK1. These results raised the possibility that NFBD1 is one of the substrates phosphorylated by PLK1. 

### PLK1-mediated phosphorylation of NFBD1 is required for M phase entry

 Because PLK1 has important roles for G2/M transition as described in the introduction, we investigated whether kinase activity of PLK1 could be responsible for M phase entry. HeLa cells were transiently transfected with an empty plasmid, the expression plasmid for FLAG-tagged PLK1 termed PLK1(WT) or with the kinase-deficient mutant form of FLAG-PLK1 termed PLK1(K82M) [28], and phosphorylation levels of histone H3 were determined by immunoblotting to reflect the mitotic index in these cells. As shown in Figure 4C, enforced expression of PLK1(WT) induced phospho-histone H3, whereas PLK1(K82M) had a marginal effect on this, suggesting that kinase activity of PLK1 is essential for the induction of the entry into M phase. 

 As mentioned above, PLK1 might have an ability to phosphorylate the PST domain of NFBD1. Therefore, we addressed if PLK1-mediated phosphorylation of NFBD1 has any function in controlling the G2/M transition. To this end, we generated expression plasmids that encompassed the PLK1-phospho-site in PST (GFP-PST) and the corresponding unphosphorylatable mutant [termed GFP-PST(T847A)] and asked if expression of these PST proteins could affect mitotic entry promoted by PLK1 (Figure 4D). We reasoned if an enforced expression of the phosphorylatable region of PST domain serves as a pseudosubstrate for PLK1, it would compete with phosphorylating endogenous PLK1 substrates including NFBD1. HeLa cells were transiently transfected with a constant amount of PLK1(WT) expression plasmid in combination with increasing amounts of GFP-PST or with GFP-PST(T847A). As observed in Figure 4E and 4F, ectopic expression of GFP-PST significantly inhibited the phosphorylation of histone H3 in a dose-dependent manner, whereas GFP-PST (T847A) had an undetectable effect on the phosphorylation levels of histone H3. 

**Figure 5:**
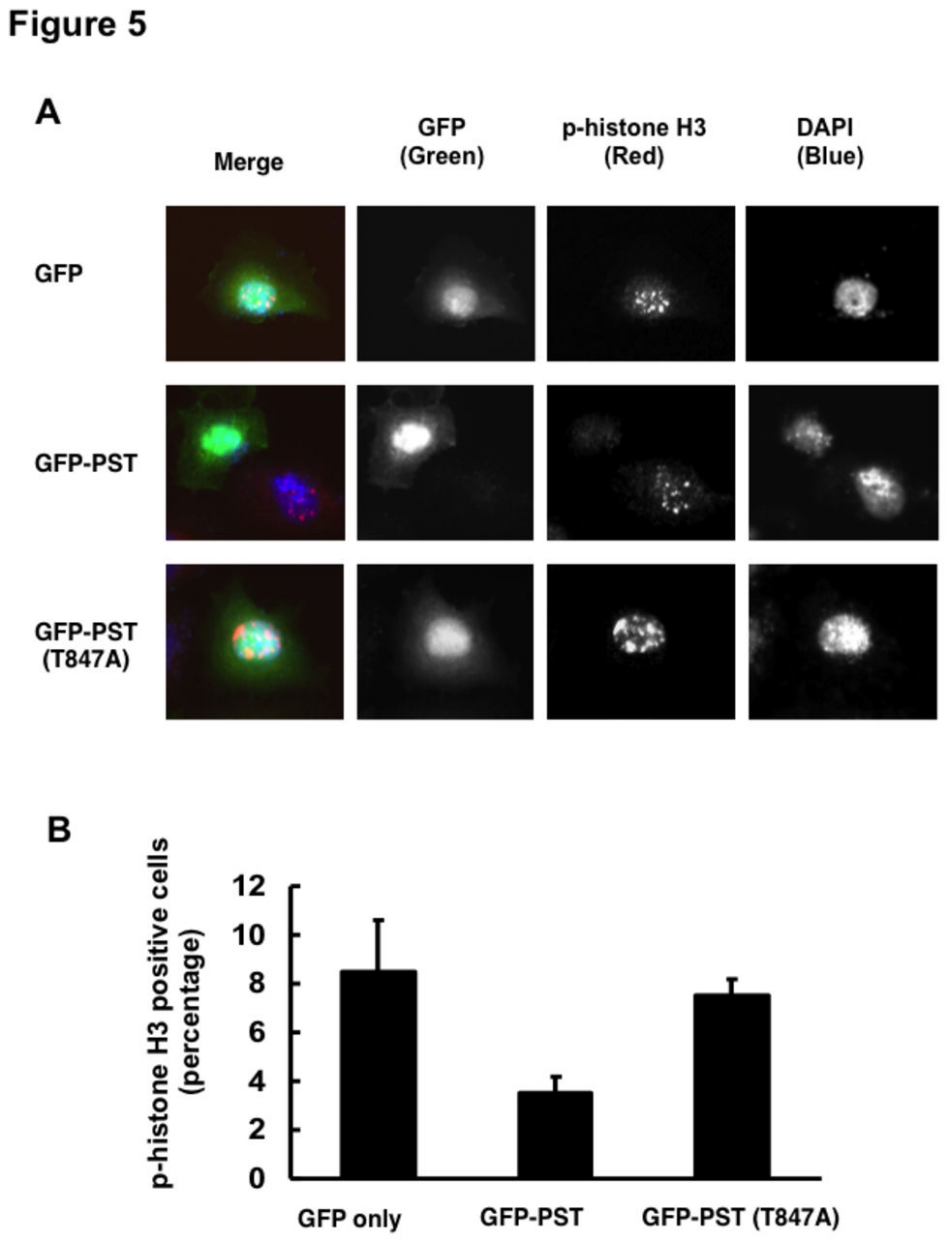
GFP-PST inhibits the phosphorylation of histone H3. (A) Indirect immunofluorescence staining. HeLa cells were transiently transfected with the indicated expression plasmids. Forty-eight hours after transfection, cells were fixed and stained with polyclonal anti-phospho-histone H3 antibody (red). Nuclear DNA was stained with DAPI (blue). Representative photos demonstrate the initial H3 phosphorylation in pericentric heterochromatin during the late G2 phase. (B) The number of GFP-positive cells (green) with phosphorylated histone H3 was scored, and the percentage of phospho-histone H3-positive cells in each column represents the mean of three independent experiments.

 To further confirm this result, we performed indirect immunofluorescence staining to evaluate whether GFP-PST or GFP-PST(T847A) could affect phospho-histone H3 status. HeLa cells were transiently transfected with the indicated expression plasmids. These cells were then fixed and stained with anti-phospho-histone H3 (Figure 5A), and the percentage of phospho-histone H3-positive cells among the GFP-positive cells were counted (Figure 5B). According to our results, enforced expression of GFP-PST significantly inhibited the phosphorylation of histone H3, whereas GFP-PST(T847A) displayed a marginal effect on the phosphorylation levels of histone H3. These experiments suggested that overexpression of PST domain interferes with M phase entry promoted by PLK1, which raises an interesting possibility that NFBD1 might be a substrate for PLK1 in controlling M phase entry.

### Knockdown of NFBD1 accelerated G2/M cell-cycle progression

**Figure 6:**
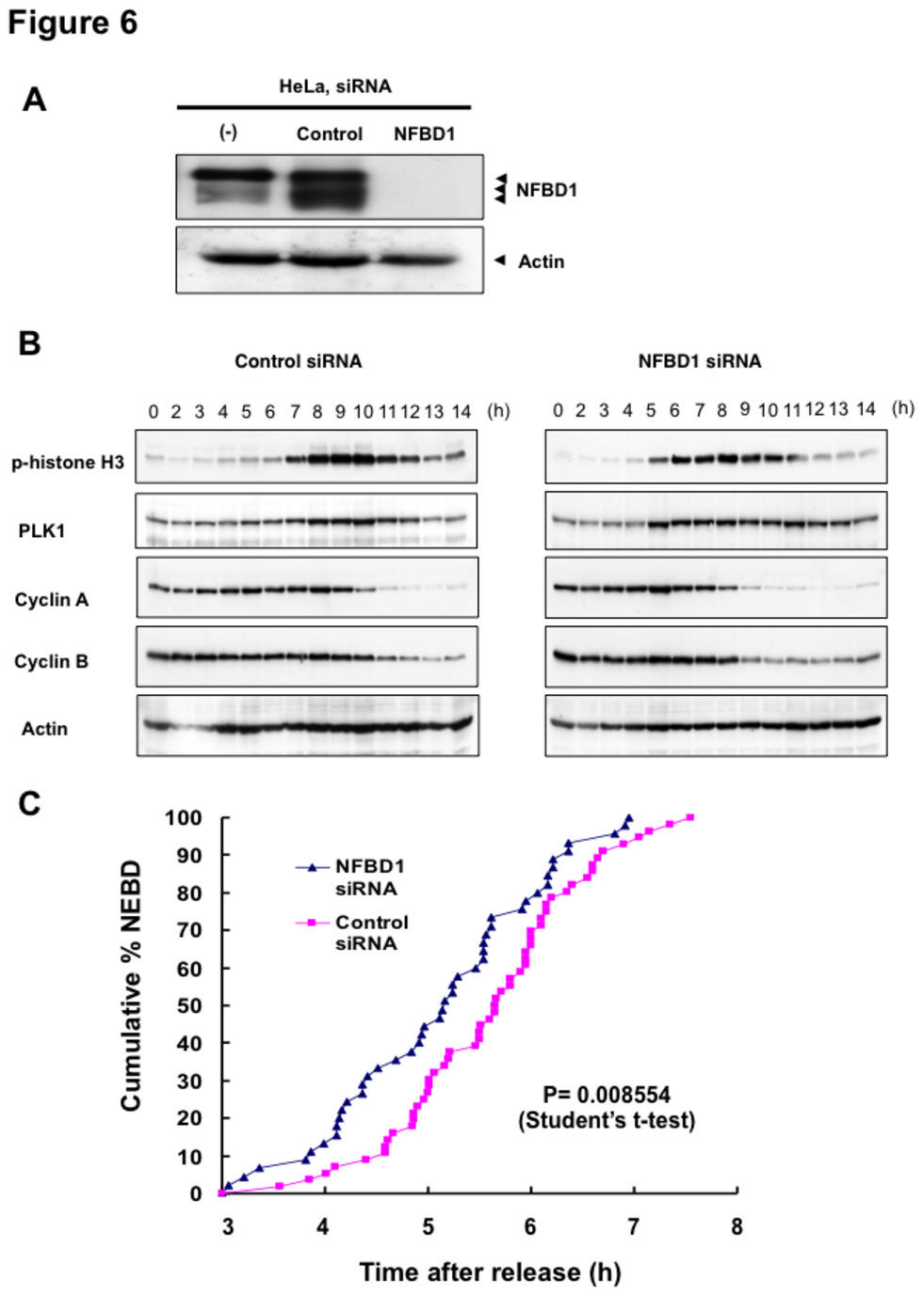
Knockdown of NFBD1 accelerates G2/M progression. (A) siRNA-mediated knockdown of NFBD1 results in a reduction of endogenous NFBD1. HeLa cells were synchronized by the double-thymidine block regimen and transfected with 10 nM of NFBD1 siRNA or control siRNA at the first release. At the time of the second release, whole cell lysates prepared from transfected cells were subjected to immunoblotting with anti-NFBD1 antibody (top panel). Western blotting for actin is shown as a control for protein loading (bottom panel). (B) Western blotting analysis of mitosis-related proteins. Whole cell lysates were prepared in NFBD1 siRNA transfected cells (right panel) or control siRNA transfected cells (left panel) at the indicated times after the second release and immunoblotted with anti-phospho-histone H3 antibody, anti-PLK1 antibody, anti-cyclin A antibody, anti-cyclin B antibody, or anti-actin antibody. (C) Cumulative percentages of NEBD-positive cells. GFP-cyclin B-expressing HeLa cells were synchronized and transfected with the indicated siRNAs. The times of NEBD were defined by the loss of a defined nuclear boundary and nuclear accumulation of cyclin B in the NFBD1 siRNA-transfected cells (n= 45) and control siRNA-transfected cells (n= 56). The significant difference was demonstrated by the Student’s *t*-test.

 To address how NFBD1 contributes to M phase entry, we next examined the possible effects on the expression of several mitosis-related proteins induced by NFBD1 knockdown. For that purpose, NFBD1 was knocked down in synchronized HeLa cells using the protocol as described in the Materials and Methods section. siRNA-mediated knockdown of NFBD1 depleted the expression of NFBD1 to undetectable levels (Figure 6A). Western blot analysis revealed that phosphorylated histone H3 and PLK1 appeared at 7 and 8 h after the second release in control siRNA transfected cells. Interestingly, both proteins were clearly detected 2 or 3 h earlier in the NFBD1 knockdown cells than in the control cells. Indeed, the early reduction of these proteins was observed in the NFBD1 knockdown cells compared with the control cells. In addition, degradation of cyclin A and cyclin B was also promoted in the cells transfected with NFBD1 siRNA (Figure 6B). These results suggest that knockdown of NFBD1 accelerates not only M phase entry but also subsequently induces M phase progression.

 To further elucidate the possible contribution of NFBD1 to entry and progression of the M phase, we asked whether knockdown of NFBD1 could affect NEBD in GFP-cyclin B-expressing HeLa cells. Times of NEBD appearance were carefully calculated in the NFBD1 knockdown cells and control cells by time-lapse microscopy. As expected, the cumulative percentages of NEBD showed that the early-onsets of NEBD were significantly observed in the NFBD1 knockdown cells compared with the control cells (*P* < 0.01) (Figure 6C). In addition, although NFBD1 knockdown resulted in the early-onset of NEBD in the cells, the time intervals between NEBD and anaphase onset were observed indefinitely, implying that checkpoint activation might be perturbed in these cells (Figure S1). Collectively, these results indicated that NFBD1 has an ability to prevent early M phase entry in a normal cell cycle.

### PLK1(T210D) expressing cells overcome decatenation checkpoint

**Figure 7:**
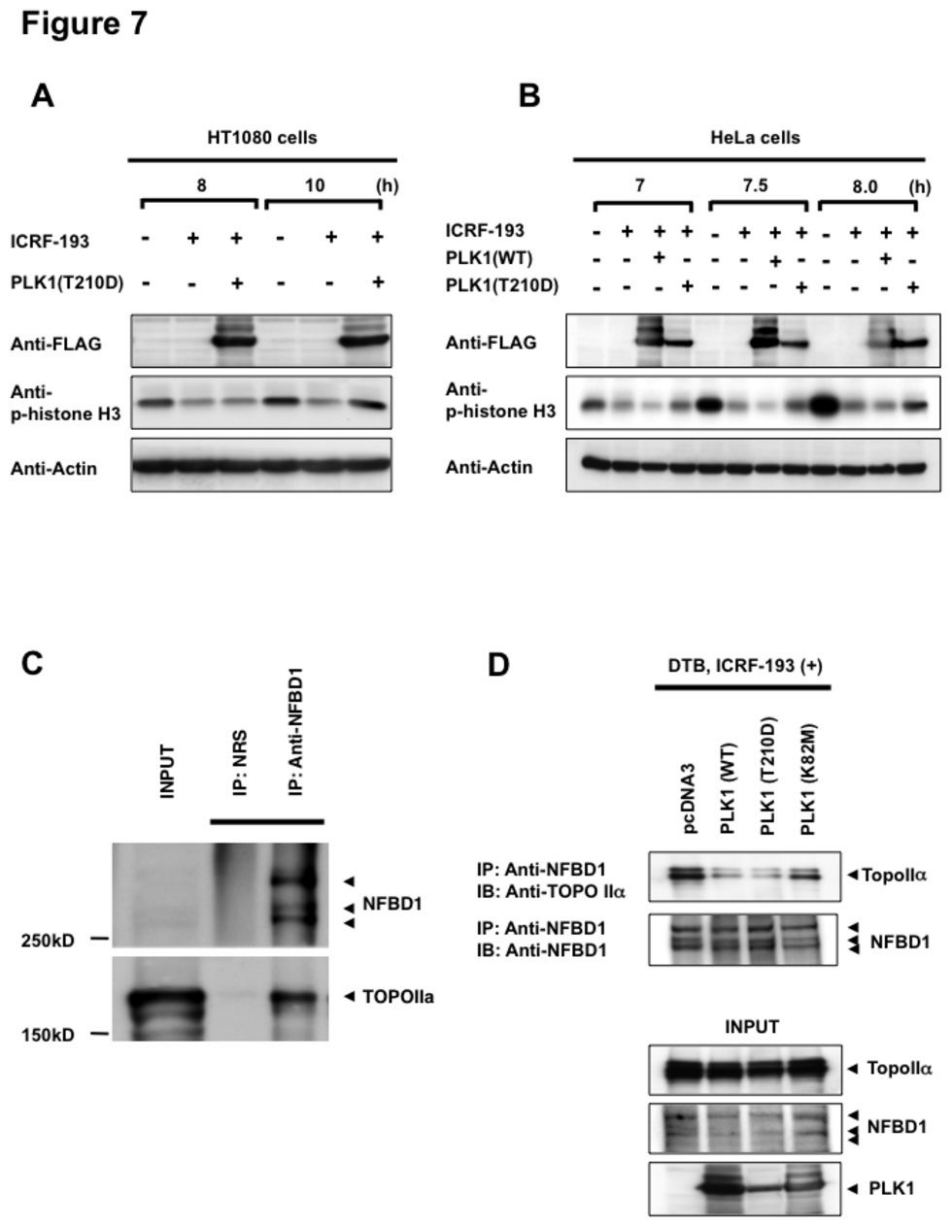
PLK1 inhibits the interaction between NFBD1 and TOPOII induced by the decatenation checkpoint. (A and B) Decatenation checkpoint assay. HT1080 cells and HeLa cells were synchronized by the double-thymidine block regimen and transfected with PLK1(WT) or PLK1(T210D) at the first release. Transfected cells were treated with the indicated concentrations of ICRF-193 from the time of the second release. Whole cell lysates were prepared at the indicated times and subjected to immunoblotting with the indicated antibodies. (C) Interaction between NFBD1 and TOPOIIα. HeLa cells were synchronized and treated with 10 μM ICRF-193. Eight hours after the second release, whole cell lysates were prepared and subjected to anti-NFBD1 immunoprecipitation followed by immunoblotting with an anti-TOPOIIα antibody. Immunoprecipitation with NRS served as a negative control. The left panel shows 1/10 loading. (D) The effects of various kinase activities of PLK1 on the ICRF-193-induced interaction between NFBD1 and TOPOIIα. HeLa cells were synchronized and transfected with PLK1(WT), PLK1(T210D), PLk1(K82M), or control plasmid at the first release. Transfected cells were treated with ICRF-193 from the time of the second release. Whole cell lysates were prepared at 8 h after the second release and immunoprecipitated and subjected to immunoblotting with the indicated antibodies.

 Consistent with previous reports, interaction between the BRCT domains of NFBD1 and TOPOIIα is required for the activation of the ICRF-193-induced decatenation checkpoint and maintenance of genomic stability in human fibrosarcoma HCT1080 cells. Notably, the phospho-mimicking T210D mutant of PLK1 can override the ICRF-193-induced checkpoint [7]. To confirm if not only HCT1080 cells but also HeLa cells can demonstrate PLK1-mediated transition from the ICRF-193-induced decatenation checkpoint, we transfected cells with PLK1(WT) or FLAG-tagged constitutively active PLK1, termed PLK1(T210D), to synchronize these cell lines and then treated the cell lines with ICRF-193. Consistent with the previous study, ICRF-193 treatment prevented phospho-histone H3 accumulation in the HCT1080 cells, suggesting G2 arrest, whereas phospho-histone H3 was clearly detected in the PLK1(T210D)-expressing HCT1080 cells, suggesting a failure to induce G2 arrest (Figure 7A). To evaluate whether similar observations could be observed in HeLa cells, PLK1(WT) and PLK1(T210D) were expressed in synchronized HeLa cells and evaluated for the effects on phospho-histone H3 accumulation in these cells. As expected, the cells with phospho-histone H3 accumulation were partially rescued from ICRF-193-induced G2 arrest in PLK1(T210D) transfected cells, but not in the PlLK1(WT)-transfected cells (Figure 7B). These results demonstrated that PLK1(T210D) could overcome the TOPOIIα-mediated decatenation checkpoint in both HCT1080 cells and HeLa cells. Because our studies suggested that PLK1 has an ability to phosphorylate NFBD1 to allow cells to enter the M phase, this observation indicated that hyper-phosphorylation of NFBD1 by PLK1 might be associated with the prevention of G2 arrest induced by the decatenation checkpoint. Increased kinase activity of PLK1 is likely to be required for completely preventing the decatenation checkpoint.

### The interaction between NFBD1 and TOPOIIα is interrupted by PLK1 in a phosphorylation-dependent manner

 To further explore the essential roles of PLK1 and its kinase activity in the decatenation checkpoint, the effects of various levels of PLK1 phospho-activities on the interaction between NFBD1 and TOPOIIα were evaluated. To this end, we transfected synchronized HeLa cells with PLK1(WT), PLK1(K82M), or PLK1(T210D) and then performed immunoprecipitation analysis in response to ICRF-193. Consistent with the previous study, TOPOIIα was detected with the anti-NFBD1 immunoprecipitates (Figure 7C). Intriguingly, low but significant levels of TOPOIIα protein were detected in the immunoprecipitates of PLK1(WT)- and PLK1(T210D)-transfected cells compared with those in the control cells and PLK1(K82M)-transfected cells (Figure 7D), suggesting that the kinase activity of PLK1 plays a critical role in the interaction between NFBD1 and TOPOIIα. Collectively, PLK1-dependent phosphorylation of NFBD1 might affect the interruption of the stable binding conformation between NFBD1 and TOPOIIα and prevent the activation of the decatenation checkpoint and subsequently induce M phase entry in mammalian cells. These findings are consistent with the hypothesis that PLK1-mediated phosphorylation of NFBD1 may relate, at least in part, to G2/M progression through the regulation of the TOPOIIα function.

## Discussion

 Prompt and accurate cellular response to DNA damage in development, genomic stability, and tumor suppression has been well recognized. Recent work in several laboratories has reported that NFBD1 is one of the members in the family of checkpoint proteins known as mediators of DNA damage signaling. Other members of these mediator proteins, such as BRCA1 and 53BP1, were initially identified as tumor suppressor gene products. Likewise, recent studies have also revealed that loss of NFBD1 may be involved in tumorigenesis [30,31]. 

 Paradoxically, our initial biochemical study revealed that NFBD1 has anti-apoptotic potential [32]. In accordance with our previous observations, it has been shown that siRNA-mediated knockdown of NFBD1 results in a significant increase in the number of apoptotic cells, and silencing of NFBD1 renders cells sensitive to irradiation [33]. Intriguingly, Xu and Stern described that NFBD1 forms a stable complex with tumor suppressor p53, suggesting that NFBD1 might modulate the pro-apoptotic function of p53 [34]. Recently, we have reported that NFBD1 inhibits ATM-dependent phosphorylation of p53 at Ser-15 during the early response to DNA damage and suppresses its pro-apoptotic activity. In contrast, NFBD1 is down-regulated in the late phase of the DNA damage response, which may induce p53-mediated apoptosis [35].

 The most likely explanation for this discrepancy may be that NFBD1 is associated with defective DNA repair. A failure of DNA repair might provide cells opportunities of genomic alteration for transformation. Our recent studies have demonstrated that the chronic effect of NFBD1 knockdown in HeLa cells caused cell-cycle arrest at the G2 phase with down-regulation of the expression levels of phospho-histone H3 and increasing endogenous DNA damage in these cells as judged from the accumulation of γ-H2AX. The vast majority of NFBD1 knockdown cells subsequently induced apoptosis; therefore, the cell proliferation rate of these cells was significantly decreased compared with the control cells [36]. However, we hypothesize that the partial viable population of NFBD1 knockdown cells might contain genomic instability and may be causally related to tumorigenesis.

 Accumulating evidence has not only revealed the functional significance of NFBD1 in response to DNA damage, but also that NFBD1 regulates the mRNA levels of several genes, such as caveolin 1 (CAV1) and caveolin 2 (CAV2) independent of IR and p53, suggesting that NFBD1 has an essential role outside the DNA damage response [37]. In this study, we identified that NFBD1 might be one of the potential substrates for PLK1. Because PLK1 is an essential mitotic kinase that regulates processes of M phase entry, our experimental design to clarify the functional relationship between PLK1 and NFBD1 was specifically focused on their contribution to G2/M transition. To date, overexpression of PLK1 has been observed in various human cancers. Therefore, NFBD1 might be exposed to PLK1-mediated hyperphosphorylation in cancer cells. This recent study provides data that allow speculation that PLK1-mediated phosphorylation of NFBD1 is associated with the decatenation checkpoint. 

 Chromosome catenation status is monitored by the decatenation checkpoint, which arrests cells at the G2 phase, delaying the onset of mitosis until sister chromatids are fully separated. Of note, the ICRF-193-induced decatenation checkpoint acts independent of the DNA damage checkpoint without the activation of ATM, CHK1, and CHK2 [38]. Therefore, our present findings that NFBD1 knockdown accelerates M phase entry might causally relate to this checkpoint. In support of our hypothesis, Li et al. reported that Plk1 directly interacts with and phosphorylates TOPOIIα at Ser^1337^ and Ser^1524^ in the S phase and requires TOPOIIα-mediated sister chromatid segregation [39]. Because NFBD1 interacts with TOPOIIα, there might be a possible cross-linkage of PLK1-mediated phosphorylation between NFBD1 and TOPOIIα, which is associated with the decatenation checkpoint to induce cells to arrest at G2/M transition. In accordance with this, our data suggested that overexpression of the PLK1 unphosphorylatable PST mutant prevented entry into the M phase. The siRNA-mediated knockdown of NFBD1 significantly accelerated early M phase entry. Therefore, PLK1-mediated phosphorylation of NFBD1 and TOPOIIα affects G2/M transition to a greater extent than S phase progression. However, our additional studies suggested that DNA synthesis was also slightly accelerated in the NFBD1 knockdown cells compared with the control cells (Figure S2). These results indicated that the monitoring of chromosome catenation status by NFBD1 most likely initializes cells from the S phase and sustains them through the G2 phase into the M phase.

 Accumulating evidence suggests that PLK1 controls recovery from the G2 phase DNA damage-induced cell-cycle arrest in mammalian cells [23,40,41]. Macůrek et al. described that aurora A promotes DNA-damage checkpoint recovery through phosphorylation of Thr 210, leading to the consequent activation of PLK1. Importantly, phosphorylation of this residue could be sufficient for initial activation of PLK1, both during an unperturbed cell cycle and during checkpoint recovery [24]. Our present observations indicate that constitutively active PLK1, PLK1(T210D), overcomes the ICRF-193-induced decatenation checkpoint and is likely to show a phenomenon similar to DNA-damage checkpoint recovery.

**Figure 8:**
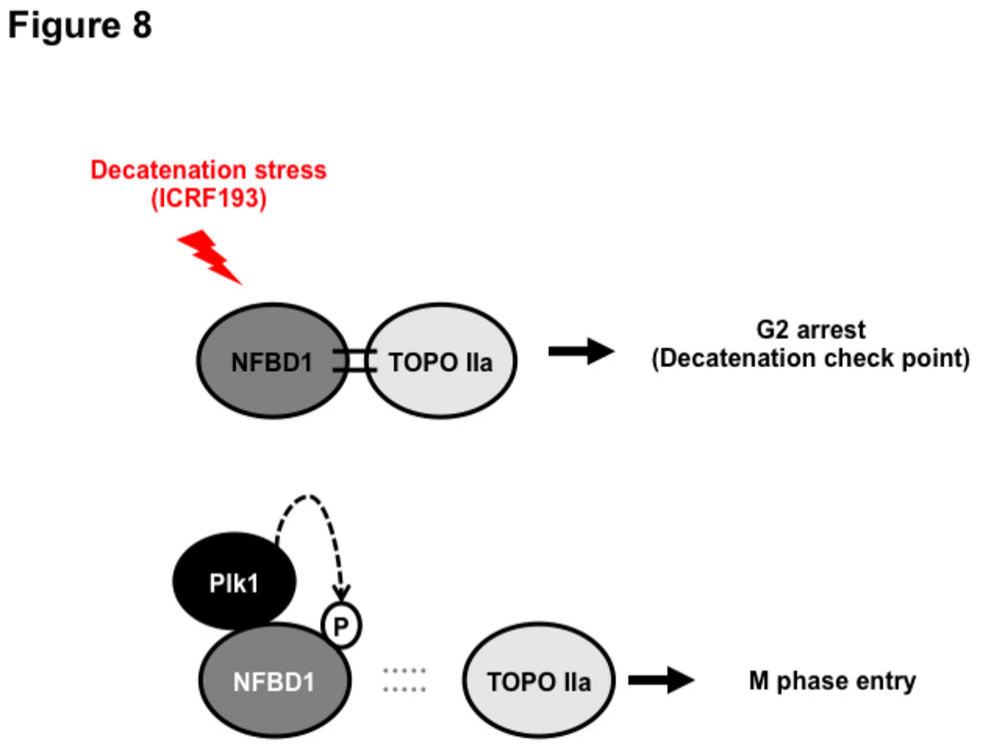
A model illustrating how NFBD1 contributes to the G2/M progression by PLK1-mediated phosphorylation.

 Finally, our data could predict possible mechanisms of how PLK1 could prevent the decatenation checkpoint. PLK1 might inhibit the interaction between NFBD1 and TOPOIIα in a phospho-dependent manner (Figure 8). We have shown that PLK1 phosphorylates NFBD1 and that NFBD1 binds TOPOIIα in PLK1 dependent manner. However, it remains to be investigated whether the phospho-NFBD1 is essential for TOPO IIα binding. Indeed, although our present results provided that both PLK1(T210D) and PLK1(WT) have an ability to prevent the interaction between NFBD1 and TOPOIIα, only PLK1(WT) failed to overcome the decatenation checkpoint. This discrepancy might be due to our experimental conditions that could at least partly involve the presence of the other types of checkpoint activation, such as DNA strand breaks. Therefore, kinase activity of PLK1(WT) might not be sufficient to allow rescue from G2 arrest in these conditions. Further studies will be required to prove that phospho-NFBD1 prevents the decatenation checkpoint to allow cells entry into the M phase. Therefore, we plan to develop a phospho-NFBD1 at the Thr 847 antibody to clarify the phospho-dependent role as well as the structural modification of the binding between NFBD1 and TOPOIIα.

 In summary, when chromatids are insufficiently decatenated, NFBD1 binds with TOPOIIα at Ser 1524 and facilitates interaction between checkpoint transducers and effectors, such as ATR and cyclin B1/CDK1, respectively. This decatenation checkpoint prevents cell-cycle progression from the G2 to M phases to maintain genomic stability. In contrast, when PLK1 is preferentially activated in cells, NFBD1 may be hyper-phosphorylated by PLK1 and released from TOPOIIα with entangled chromatins. Therefore, these cells may have a defect of the decatenation checkpoint that induces improper sister chromatid segregation leading to genomic instability and tumorigenes

## Materials and Methods

### Cell culture and transfection

 Human cervical carcinoma HeLa cells, African green monkey kidney COS7 cells, and human fibrosarcoma HT1080 cells were grown in Dulbecco’s modified Eagle’s medium supplemented with 10% heat-inactivated fetal bovine serum, 50 　μg/ml penicillin, and 50 μg/ml streptomycin (Invitrogen). For transfection, cells were transfected with the indicated expression plasmids using FuGENE HD transfection reagent (Roche Applied Science) according to the manufacturer's instructions. 

### Cell synchronization

 Cells were synchronized in the late G1 phase by a double-thymidine block. In brief, cells were treated with 1 mM of thymidine (Sigma) for 24 h, released for 8 h (henceforth, the first release), and then treated again with thymidine for 16 h. After two washes with PBS, the cells were cultured in normal medium (henceforth, the second release). 

### Immunoblotting and immunoprecipitation

 Immunoblotting and immunoprecipitation were performed as previously described [35]. The primary antibodies used for this study were as follows: monoclonal anti-FLAG (M2, Sigma), monoclonal anti-Plk1 (PL2 and PL6, Zymed Laboratories), monoclonal anti-GFP (1E4, Medical and Biological Laboratories), monoclonal anti-cyclin A (BF683, Cell Signalling), monoclonal anti-cyclin B (18, BD Bioscience), monoclonal anti-topoisomerase IIα (Ki-S1, Boehringer Mannheim) polyclonal anti-NFBD1 [35], polyclonal anti-phospho-histone H3 at Ser-10 (Cell Signaling), and polyclonal anti-actin (20-33, Sigma) antibodies. 

### Indirect immunofluorescence microscopy

 HeLa cells were fixed with 3.7% formaldehyde, permeabilized with 0.1% Triton X-100, and then blocked with 3% bovine serum albumin. After blocking, cells were simultaneously incubated with monoclonal anti-Plk1 (F8, Santa Cruz Biotechnology) and polyclonal anti-NFBD1 antibodies followed by incubation with Alexa Fluor 568-conjugated goat anti-mouse IgG and Alexa Fluor 488-conjugated goat anti-rabbit IgG (Molecular Probes). Cell nuclei were stained with DAPI. The specific fluorescence was captured on a Zeiss Imager M1 microscope equipped with epifluorescence and a Photometrics Cool Snap HQ CCD camera driven by MetaMorph software (Universal Imaging). 

### Flow cytometry

 After the addition of serum, floating and attached cells were collected and washed in ice-cold PBS. Cells were treated with 500 μg/ml of RNase A (Sigma) and subsequently stained with 50 μg/ml of propidium iodide (PI, Sigma) with 0.2% Triton X-100 for 30 min at room temperature. Subsequently, DNA content indicated by PI staining was analyzed using FACSCalibur flow cytometry of 10,000 cells (Becton Dickinson). 

### Plasmid construction

 The construction of wild-type PLK1 as well as mutant forms of PLK1, FHA, PST, and BRCT expression vectors has been described previously [28,35]. The T847A mutation was introduced into the GST-PST(806-881) using a QuikChange II XL Site-Directed Mutagenesis kit (STRATAGENE) according to the manufacturer’s instructions. The specific primers used are as follows: 5′-CAGACAGATGTGGCAGGAGAGGAAG-3′ and 5′-CTTCCTCTCCTGCCACATCTGTCTG-3′ (underlined segment encodes Ala at amino acid position 847). Nucleotide sequences of the PCR products were determined to verify the presence of the desired mutation and the absence of random mutations. 

### RNA interference

 To knock down endogenous NFBD1, HeLa cells were transiently transfected with 10 nM of the chemically synthesized siRNA targeting NFBD1 (003506-06, Dharmacon) or with control siRNA (Invitrogen) using Lipofectamine™ RNAiMAX (Invitrogen) at the time of the first release, followed by the addition of thymidine. Whole cell lysates were prepared at the indicated times after the second release and subjected to immunoblotting or immunoprecipitation.

### GST pull-down assay

 A cDNA fragment encoding for the COOH-terminal BRCT domains of NFBD1 was subcloned into the appropriate restriction sites of pGEX-5X-1 (Amersham Biosciences). GST-BRCT fusion protein was expressed in *E. coli* DH5and purified using glutathione Sepharose beads (Amersham Biosciences). The indicated COOH-terminal deletion mutants of PLK1 were radiolabeled *in vitro* using the T_N_T QuickCoupled transcription/translation system (Promega) in the presence of [^35^S]methionine and incubated with GST-BRCT fusion protein for 2 h at 4°C. After the addition of 30 μl of glutathione Sepharose beads, the reaction mixtures were incubated for 1 h a 4°C. The beads were then collected by brief centrifugation and washed three times with binding buffer containing 50 mM Tris-HCl, pH 7.5, 150 mM NaCl, 0.1% Nonidet P-40, and 1 mM EDTA. The ^35^S-labeled bound proteins were eluted by 2X SDS sample buffer and separated by 10% SDS-PAGE. After electrophoresis, the gel was stained with CBB followed by autoradiography. 

### *In vitro* kinase reaction

 GST, GST-PST, or GST-PST(T847A) was incubated with the active form of PLK1 (CycLex) in a solution containing 40 mM MOPS-NaOH, pH 7.0, 1 mM EDTA, and 25 mM sodium acetate in the presence of [γ-^32^P]ATP at 30°C for 30 min. After incubation, the reaction mixtures were separated by SDS-PAGE. The gel was then dried and subjected to autoradiography.

### Live cell imaging analysis

 GFP-cyclin B1-expressing HeLa cells were grown in chambered coverslips (Laboratory-Tek; Thermo Fisher Scientific) and synchronized and then transfected with NFBD1 siRNA or control siRNA. One hour before imaging, the medium was changed to prewarmed CO_2_-independent medium without phenol red (Invitrogen), and the chamber lids were sealed with silicone grease. DNA was stained with 0.1 μg/ml Hoechst 33342. Recordings were performed after 3 h from the second release at 37°C using a custom-built temperature-controlled incubator. Time-lapse images were collected at 3-min intervals with a 60 × 1.518 NA Plan Apochromat oil objective lens (Carl Zeiss, Inc.) mounted on an inverted microscope (IX-71; Olympus) equipped with a charge-coupled device camera (CoolSNAP HQ; Photometrics) that was driven by softWoRx 3.6.0 software (Applied Precision, LLC). Data analysis was performed using ImageJ version 1.37 software (National Institutes of Health).

### Decatenation checkpoint assay

 Cells were synchronized by double-thymidine block and treated with the indicated concentration of ICRF-193 (Sigma) from the time of the second release. Whole cell lysates were prepared at the indicated times and subjected to immunoblot or immunoprecipitation.

## Supporting Information

Figure S1 **siRNA-mediated knockdown of NFBD1 leads to an indefinite time interval between NEBD and anaphase onset in cells.** GFP-cyclin B-expressing HeLa cells were synchronized and transfected with the indicated siRNAs as described in the Materials and Methods section. The times of anaphase onset were defined by sister chromatid segregation. The time intervals between NEBD1 and anaphase onset were measured in the NFBD1 siRNA-transfected cells (n= 45) or control siRNA-transfected cells (n= 56) and indicated as vertical lines.
(TIFF)

Figure S2 **siRNA-mediated knockdown of NFBD1 slightly accelerated S phase progression.** S phase progression analysis. HeLa cells were synchronized by the double-thymidine block regimen and transfected with NFBD1 siRNA and control siRNA at the time of the first release. At the indicated times after the second release, cells were stained with PI and subjected to FACS analysis.
(TIFF)
