# Supplementary figures and images for: NFBD1/MDC1 Is Phosphorylated by PLK1 and Controls G2/M Transition through the Regulation of a TOPOIIα-Mediated Decatenation Checkpoint
Source: PLoS One. 2013 Dec 11;8(12):e82744. doi: 10.1371/journal.pone.0082744 (PMC3859618; doi:10.1371/journal.pone.0082744)

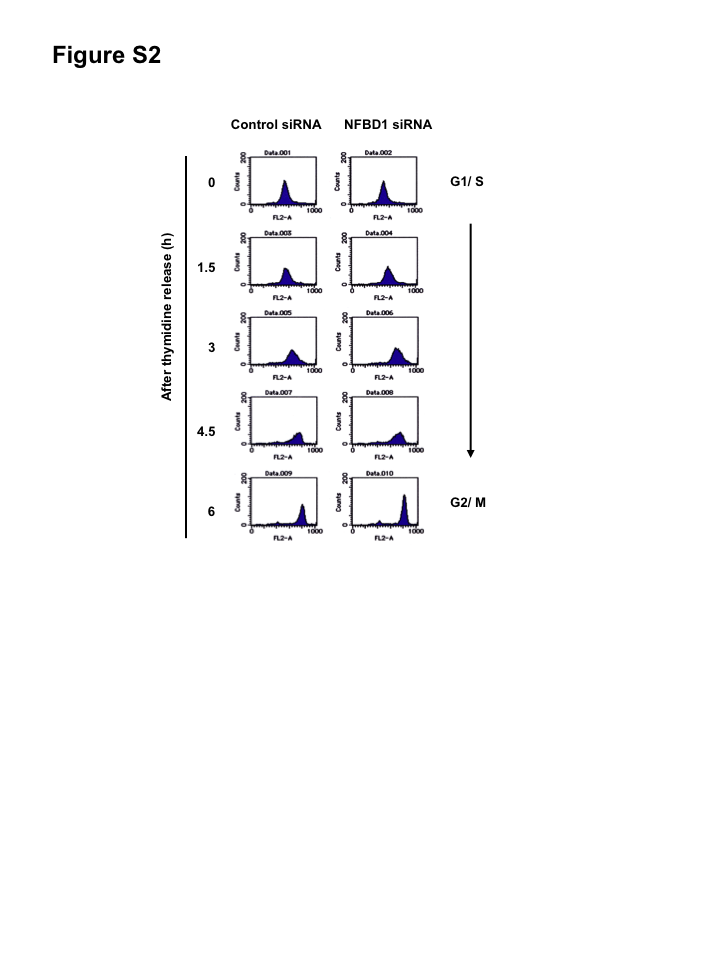

Supplement: Figure S2 — siRNA-mediated knockdown of NFBD1 slightly accelerated S phase progression. S phase progression analysis. HeLa cells were synchronized by the double-thymidine block regimen and transfected with NFBD1 siRNA and control siRNA at the time of the first release. At the indicated times after the second release, cells were stained with PI and subjected to FACS analysis. (TIFF) [file pone.0082744.s002.tiff]
